# Assessment of an innovative synthetic matrix for enhanced cell preservation: evaluating its clinical utility and impact on diagnostic precision in thyroid fine-needle aspiration cytology

**DOI:** 10.1186/s12902-025-02147-0

**Published:** 2026-02-09

**Authors:** Diana-Raluca Streinu, Octavian Constantin Neagoe, Andreea Bena, Dana-Liana Stoian

**Affiliations:** 1 Department of Doctoral Studies, Victor Babes University of Medicine and Pharmacy, Timisoara, Romania; 2 Center of Molecular Research in Nephrology and Vascular Disease, Faculty of Medicine, Victor Babes University of Medicine and Pharmacy, Timisoara, Romania; 3 Second Clinic of General Surgery and Surgical Oncology, Timisoara Municipal Emergency Clinical Hospital, Timisoara, Romania; 4 2nd Department of Internal Medicine, Victor Babes University of Medicine and Pharmacy, Timisoara, Romania; 5 First Surgery Clinic, “Pius Brinzeu” Clinical Emergency Hospital, Timisoara, Romania; 6 DR.D Medical Center, Timisoara, Romania

**Keywords:** Cytomatrix, FNAC, Thyroid nodules, Smears, Cytology

## Abstract

**Background:**

Thyroid nodules are common clinical findings that often require fine-needle aspiration cytology (FNAC) for evaluation. This study aimed to assess the clinical value of Cytomatrix, a commercially available synthetic 3D matrix designed to improve preservation of cytological samples, in enhancing the diagnostic accuracy of thyroid fine-needle aspiration cytology in a standard clinical setting.

**Methods:**

A prospective study was conducted at Dr. D Medical Center (Timișoara) between January 2023 and January 2025, involving 80 patients with high-risk thyroid nodules classified as EU-TIRADS 4 or higher (European Thyroid Imaging Reporting and Data System). Each patient underwent FNAC with samples collected using both the conventional smear technique and the Cytomatrix method. Both sample types were processed under standard laboratory conditions with minimal technician involvement. Smears were classified according to the 2023 Bethesda System, and Cytomatrix samples were evaluated using a corresponding Bethesda classification. The diagnostic outcomes of both approaches were compared with each other and against the final histopathological results.

**Results:**

Cytomatrix exhibited superior diagnostic performance, achieving a sensitivity of 78.2%, specificity of 96.4%, positive predictive value (PPV) of 90%, and overall accuracy of 91.2%. In comparison, the conventional smear method demonstrated a sensitivity of 69.5%, specificity of 94.7%, PPV of 84.2%, and accuracy of 87.5%. The two methods showed a concordance rate of 76.2%. Cytomatrix notably refined Bethesda classifications by both upgrading and downgrading smear-based diagnoses, aligning more closely with histopathology findings.

**Conclusion:**

Cytomatrix represents a promising addition to traditional FNAC in clinical settings characterized by minimal resource use and reduced technician intervention. Its improved diagnostic accuracy and streamlined processing may enhance thyroid nodule evaluation, contributing to better patient management outcomes.

**Clinical trial number:**

Not applicable.

## Introduction

Fine-needle aspiration cytology (FNAC) remains the gold standard in the diagnosis of thyroid nodular disease, widely acknowledged for its diagnostic reliability [1]. The rising incidence of thyroid cancer can be attributed to a combination of improved diagnostic techniques and changes in environmental and lifestyle factors [2–4]. FNAC is a vital tool in thyroid disease management, allowing clinicians to accurately differentiate between benign and malignant lesions with minimal invasiveness.

Ultrasound guidance is employed by specialists to identify suspicious thyroid lesions. A small-gauge needle is used to extract cellular material from the nodule, which is then smeared onto slides for cytological analysis [5]. The results are evaluated using the 2023 Bethesda System for Reporting Thyroid Cytopathology, which classifies findings into six categories: (Bethesda I) nondiagnostic, (Bethesda II) benign, (Bethesda III) atypia of undetermined significance, (Bethesda IV) follicular neoplasm, (Bethesda V) suspicious for malignancy (SFM), and (Bethesda VI) malignant [6].

Despite advancements in diagnostic techniques, fine-needle aspiration cytology (FNAC) still has limitations, often yielding inconclusive results, particularly due to suboptimal sample collection and processing [7]. A significant challenge in clinical decision-making is the frequent occurrence of Bethesda Category III (atypia of undetermined significance), which creates diagnostic ambiguity and requires further testing [8, 9]. Additionally, the smearing process, essential for slide preparation, can distort cellular architecture, altering the performance of ancillary tests that could help clarify the diagnosis, especially in indeterminate cases [10, 11].

In a prior study, we assessed the performance of Cytomatrix, a commercial synthetic matrix, which demonstrated significant potential in enhancing diagnostic accuracy in experimental settings performing FNAC on excised thyroid glands, with minimal technician intervention and requiring only limited resources [11]. Developed by UCS Diagnostic Srl in collaboration with Campus Bio-Medico University of Rome (Joint ownership Patent N °: 102016000111352), Cytomatrix is an innovative technology for preserving cells, maintaining the structural integrity of thyroid cells from FNAC samples within a 3D synthetic matrix. This method allows the deposited cytology to closely resemble histological tissue, reducing the need for extensive technician intervention and improving diagnostic precision. The preserved matrix is stable for indefinite storage and can support various additional diagnostic techniques, such as immunohistochemistry (IHC), fluorescence in situ hybridization (FISH), and molecular assays. Furthermore, it requires less cellular material than traditional methods, enhancing sample collection while preserving essential material for subsequent testing [12].

This study explores the potential of incorporating Cytomatrix into clinical FNAC procedures to improve diagnostic accuracy and procedural efficiency. By enhancing cell preservation and sample integrity, Cytomatrix may reduce the incidence of inconclusive or inadequate samples, thereby minimizing the need for repeat aspirations [11, 12]. This, in turn, can simplify workflow for clinicians, reduce patient discomfort, and speed up the diagnostic process. Ultimately, by providing clearer and more reliable cytological results, this approach has the potential to facilitate earlier and more precise therapeutic decisions, leading to improved patient outcomes.

## Materials and methods

This prospective study was conducted at Dr. D Medical Center in Timișoara between January 2023 and January 2025. The inclusion criteria were carefully designed to ensure the reliability of the comparative analysis. Patients eligible for the study had thyroid nodules classified as at least EU-TIRADS 4, according to the European TIRADS classification [13], as identified through ultrasound, indicating a higher risk of malignancy. Only individuals who underwent FNAC using both conventional smears and Cytomatrix, followed by thyroidectomy, were included, ensuring that all cases had corresponding histopathological outcomes for direct comparison.

Exclusion criteria were defined to maintain the integrity of the analysis. Patients who did not undergo thyroidectomy following thyroid FNAC were excluded due to the lack of histopathological confirmation; cases in which the histopathological result was unavailable were also excluded. Bethesda I cases were not included as these represent nondiagnostic results that provide no clear basis for comparison with final histopathological outcomes [6]. Although Bethesda I cases were excluded from diagnostic performance calculations, the non-diagnostic rate was assessed across the full cohort prior to exclusion to transparently evaluate sampling adequacy. Cases lacking results from both methods or corresponding histopathology were excluded to ensure valid and balanced comparisons. Nevertheless, the non-diagnostic rate remains an important metric and is discussed accordingly.

Ultimately, based on the outlined inclusion criteria, the study included 80 patients aged 23 to 74 years old, comprising 64 females and 16 males, all of whom underwent thyroidectomy following FNAC.

All participants provided informed consent, ensuring compliance with the ethical guidelines outlined in the Declaration of Helsinki.

FNAC was performed under real-time ultrasound guidance by an experienced clinician to ensure precise targeting of the suspicious nodules. A 25-gauge needle attached to a 10 ml syringe was used, employing a hybrid approach in which both capillary (non-aspiration) and aspiration methods were applied simultaneously during the same needle pass. As the needle was inserted into the nodule, initial capillary sampling was performed, followed by gentle aspiration to enhance cellular yield without compromising sample quality [14].

**Fig. 1:**
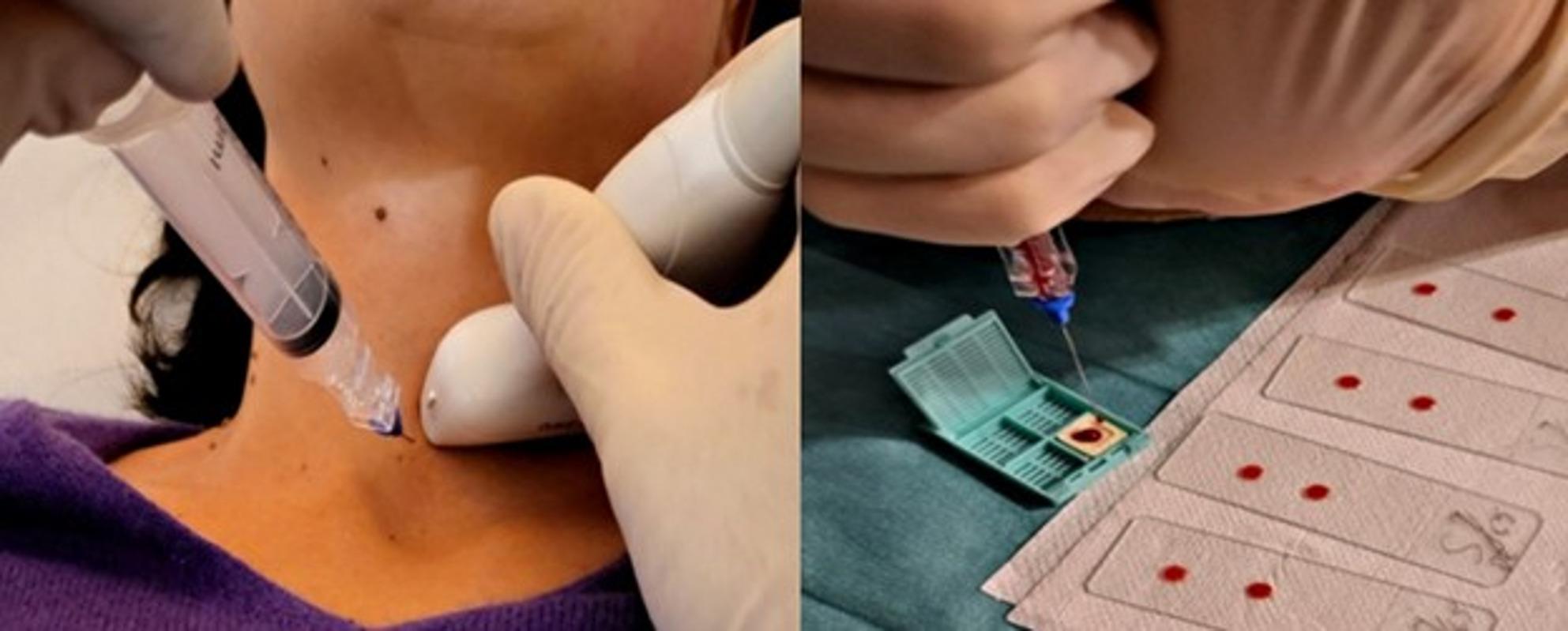
Sample collection (left) and deposition (right). FNAC procedure representing ultrasound-guided aspiration (Left). Deposition of sample onto Cytomatrix and conventional slides (Right)

Following the aspiration, the collected material was processed in two ways: the primary sample was used to prepare four conventional smears for standard cytological evaluation. From the same syringe, 1–2 drops of the remaining material were carefully deposited onto the Cytomatrix. This dual-sample approach allowed for a direct comparison between conventional smears and the Cytomatrix. By using samples from the same collected material for both methods, variability in sample origin was significantly minimized, ensuring consistency in the comparative analysis of diagnostic accuracy and quality of cytological assessment. The described steps are illustrated in Fig. 1.

The four conventional smears were immediately fixed with a cytological fixative spray to prevent air-drying artifacts and were subsequently processed using standard cytological staining techniques, to allow optimal visualization of cellular morphology and nuclear detail. To enhance smear quality and diagnostic reliability, two drops of aspirated material were placed on each of the four slides. This approach was chosen to promote even cellular distribution, reduce the risk of cell overlapping and clumping, and minimize obscuring elements such as blood or mucus. By spreading the sample more uniformly across multiple slides, this technique increases the likelihood of obtaining diagnostically useful areas with well-preserved cellular details, which are critical for accurate cytological evaluation. The Cytomatrix cassette, containing the synthetic matrix that holds the collected material, was immersed in formalin for at least 12 h for proper fixation, followed by embedding in paraffin, sectioning into 10-micron slices with a microtome, and staining with hematoxylin and eosin (H&E) for detailed cytological examination.

Conventional smears were analyzed and interpreted using the 2023 Bethesda System [6]. However, due to Cytomatrix’s lack of standardization in thyroid FNAC, a version that mirrors the 2023 Bethesda System was used for Cytomatrix analysis.

All cytological slides and Cytomatrix were reviewed and classified by a single certified cytologist.

Bethesda III (AUS) cases were considered cytologically indeterminate but considered as more likely benign, based on literature reporting a malignancy risk of 5–30% [15, 16]. For the purposes of statistical calculations—including sensitivity, specificity, predictive values, and McNemar’s test—Bethesda categories II and III were grouped as benign, while categories IV, V, and VI were grouped as malignant. This binary classification was adopted to facilitate clearer assessment of diagnostic performance, consistent with methodologies used in prior studies [17]. However, we acknowledge that Bethesda III represents a diagnostically ambiguous category with variable risk, and its clinical interpretation is more nuanced. Accordingly, these cases were included in the analysis and classified according to their final histopathological outcomes: those with benign histology were considered true negatives, and those with malignancy were treated as false negatives. This approach allowed for robust statistical evaluation while still reflecting the inherent uncertainty of Bethesda III cases, which was further addressed in the results and discussion.

Histopathological examination of thyroidectomy specimens was conducted in an independent laboratory according to the WHO guidelines, serving as the gold standard for validating the cytological findings [18]. The results from conventional smears and Cytomatrix were compared with each other and the final histopathological diagnosis to assess concordance and evaluate Cytomatrix’s potential to improve FNAC diagnostic accuracy in clinical practice. This evaluation was conducted using minimal resources, deliberately avoiding specialized equipment and additional testing to assess its effectiveness under standard conditions.

## Results

### Non-diagnostic rates

A total of 14 cases were classified as non-diagnostic (Bethesda I) and excluded from the final analysis, as no corresponding histopathological outcomes were available. Among these, 9 were non-diagnostic on conventional smears. Of those 9 cases, 5 were also non-diagnostic on Cytomatrix, while the remaining 4 were deemed adequate by Cytomatrix but were excluded to preserve consistency in the comparison. Importantly, there were no instances in which a case was non-diagnostic on Cytomatrix but diagnostic on smears.

Out of the 80 cases analyzed, 61 (76.25%) showed concordant Bethesda categories between the two methods.

- **Bethesda II (Benign)**: 38 cases were consistently classified as benign by both methods, and confirmed benign by histopathology.
- **Bethesda III (Atypia of Undetermined Significance)**: 11 cases were classified as Bethesda III. Of these, 8 were confirmed to be benign, while 3 were malignant.
- **Bethesda V (Suspicious for Malignancy)**: All 12 cases classified as Bethesda V were confirmed malignant.

Out of the 80 cases, 19 (23.75%) showed discordance between the two methods. The specific findings for these discordant cases are as follows:

- **Bethesda II to Bethesda III**: 2 cases were upgraded from Bethesda II to Bethesda III on Cytomatrix, both of which were confirmed benign.
- **Bethesda II to Bethesda V**: 2 cases were upgraded from Bethesda II to Bethesda V, with 1 confirmed malignant and the other benign.
- **Bethesda III to Bethesda V**: 2 cases were upgraded from Bethesda III to Bethesda V, both of which were confirmed malignant.
- **Bethesda III to Bethesda IV**: 1 case was upgraded from Bethesda III to Bethesda IV and later confirmed benign.
- **Bethesda V to Bethesda VI**: 3 cases were upgraded from Bethesda V to Bethesda VI, all of which were confirmed malignant.

Cytomatrix downgraded 9 cases (11.25%) in total. The details of these downgrades are as follows:

- **Bethesda III to Bethesda II**: 5 cases were downgraded from Bethesda III to Bethesda II, with 4 confirmed benign and 1 confirmed malignant.
- **Bethesda IV to Bethesda III**: 3 cases were downgraded from Bethesda IV to Bethesda III, with 1 confirmed malignant and 2 confirmed benign.
- **Bethesda V to Bethesda II**: 1 case was downgraded from Bethesda V to Bethesda II and was later confirmed benign.

### Bethesda III results summary

Cytomatrix and smears agreed on a Bethesda III diagnosis in 11 cases, with 8 confirmed benign and 3 malignant by histology. In 3 discordant cases, Cytomatrix upgraded smears from Bethesda III to higher categories (IV or V), correctly identifying 2 malignancies. In 5 cases, Cytomatrix downgraded smears from Bethesda III to Bethesda II; 4 were benign and 1 was malignant. Additionally, Cytomatrix downgraded 3 smear-based Bethesda IV cases to Bethesda III, of which 2 were benign and 1 malignant.

The data summarizing the concordant and discordant cases between conventional smears and Cytomatrix, along with the corresponding histopathological outcomes, is presented in Table 1.

**Table 1:** Concordance between cytomatrix and smears by Bethesda classification and histopathology outcomes

Concordant Cases (61 Total)			
Smears (Bethesda Category)	Cytomatrix (Bethesda Category)	Total Cases	Histopathology Outcome
Bethesda II	Bethesda II	38	Benign
Bethesda III	Bethesda III	11	8 Benign, 3 Malignant
Bethesda V	Bethesda V	12	Malignant
Total Concordant Cases	—	61	46 Benign, 15 Malignant

Discordant Cases (19 Total)			
Smears (Bethesda Category)	Cytomatrix (Bethesda Category)	Total Cases	Histopathology Outcome
Bethesda II	Bethesda III	2	Benign
Bethesda II	Bethesda V	2	1 Benign, 1 Malignant
Bethesda III	Bethesda V	2	Malignant
Bethesda III	Bethesda IV	1	Benign
Bethesda V	Bethesda VI	3	Malignant
Bethesda III	Bethesda II	5	4 Benign, 1 Malignant
Bethesda IV	Bethesda III	3	2 Benign, 1 Malignant
Bethesda V	Bethesda II	1	Benign
**Total Discordant Cases**		**19**	**11 Benign**,** 8 Malignant**

**McNemar’s** test was performed to evaluate diagnostic discordance between Cytomatrix and conventional smears, using final Bethesda category interpretations. The test result was not statistically significant (χ² = 0.00, *p* = 1.000), indicating no systematic difference in diagnostic classification between the two methods. This suggests a balanced pattern of disagreement, with neither technique consistently over- or under-classifying relative to the other.

A formal analysis was conducted to determine whether diagnostic reclassifications by Cytomatrix (upgrades or downgrades relative to conventional smears) were associated with malignancy on final histopathology.

**Fisher’s exact test** showed no statistically significant relationship between whether a case was upgraded or downgraded by Cytomatrix and the final histopathological outcome (odds ratio = 2.63, *p* = 0.596). While the test did not confirm a definitive pattern, a descriptive trend was observed: Cytomatrix downgrades were more frequently associated with benign outcomes. This may reflect its potential utility in correcting overcalls and improving diagnostic precision in borderline cases. However, the lack of statistical significance likely reflects the small number of discordant cases in this subset.

Table 2 presents the breakdown of thyroid FNAC cases processed with Cytomatrix, classified by the mirrored Bethesda system. It correlates the Bethesda categories with the final histopathological outcomes, distinguishing between benign and malignant diagnoses. This summary highlights Cytomatrix’s diagnostic performance across various risk categories.

**Table 2:** Distribution of thyroid FNAC cases processed with cytomatrix, showing bethesda categories and corresponding histopathological outcomes

Bethesda Category	Total Cases Identified (Cytomatrix)	Benign Histopathology	Malignant Histopathology
Bethesda II	44	43	1
Bethesda III	16	12	4
Bethesda IV	1	1	0
Bethesda V	16	1	15
Bethesda VI	3	0	3
**Total**	**80**	**57**	**23**

The sensitivity of Cytomatrix was 78.2%, meaning it identified 78.2% of the malignant cases. The specificity was 96.4%, indicating that Cytomatrix identified 96.4% of the benign cases. The accuracy was 91.2%, showing that Cytomatrix correctly identified 91.2% of the cases overall. The positive predictive value (PPV) was 90%, meaning that 90% of the cases classified as malignant by Cytomatrix were confirmed malignant by histopathology.

Table 3 presents the distribution of thyroid FNAC cases classified using the conventional smear-based method according to the Bethesda system. The table details the total number of cases identified in each Bethesda category and their corresponding histopathological outcomes, differentiating between benign and malignant diagnoses. This data provides insight into the diagnostic correlation between the cytological analysis and definitive histopathology.

**Table 3:** Bethesda categories and histopathology of thyroid FNAC processed by conventional smear

Bethesda Category	Total Cases Identified (Smears)	Benign (Histopathology)	Malignant (Histopathology)
Bethesda II	42	41	1
Bethesda III	19	13	6
Bethesda IV	3	2	1
Bethesda V	16	1	15
**Total**	**80**	**57**	**23**

The sensitivity of the smear-based method was 69.5%, meaning it correctly identified 69.5% of the malignant cases. The specificity was 94.7%, indicating that the slides correctly identified 94.7% of the benign cases. The accuracy was 87.5%, showing the slides correctly identified 87.5% of the cases overall. The positive predictive value (PPV) was 84.2%, meaning that 84.2% of the cases classified as malignant by the slides were confirmed malignant by histopathology.

Of the 80 cases analyzed, 61 showed concordance between the results obtained using Cytomatrix and traditional smear-based diagnoses, yielding a concordance rate between the two methods of 76.2%.

A comparative evaluation of sensitivity, specificity, positive predictive value (PPV), and accuracy for conventional slide smears and the Cytomatrix is presented in Table 4.

**Table 4:** Comparative analysis of diagnostic performance metrics between traditional smears and cytomatrix

Metric	Cytomatrix (%) [95% CI]	Smears (%) [95% CI]
Sensitivity	78.26% [58.1–90.34]	69.57% [49.13–84.4]
Specificity	96.49% [88.08–99.03]	94.74% [85.63–98.19]
Accuracy	91.25% [83.02–95.7]	87.5% [78.5–93.07]
PPV	90.0% [69.9–97.21]	84.21% [62.43–94.48]
NPV	91.67% [81.93–96.39]	88.52% [78.16–94.33]

The diagnostic performance of the Cytomatrix technique and conventional smears was evaluated independently against final histopathological results across the 80 cases. Cytomatrix demonstrated an area under the receiver operating characteristic (ROC) curve (AUC) of 0.943 (95% CI: 0.867–0.982), while conventional smears demonstrated an AUC of 0.921 (95% CI: 0.839–0.970). Both methods showed excellent diagnostic accuracy, with results statistically significant (*p* < 0.0001). A pairwise comparison of the ROC curves revealed no significant difference between the two methods. The difference in AUCs was 0.0214 (SE = 0.0397; 95% CI: − 0.0565 to 0.0993), with a z statistic of 0.537 and a corresponding p-value of 0.5910. While this difference was not statistically significant, the slightly higher AUC for Cytomatrix may still hold clinical relevance in certain diagnostic contexts.

**Fig. 2:**
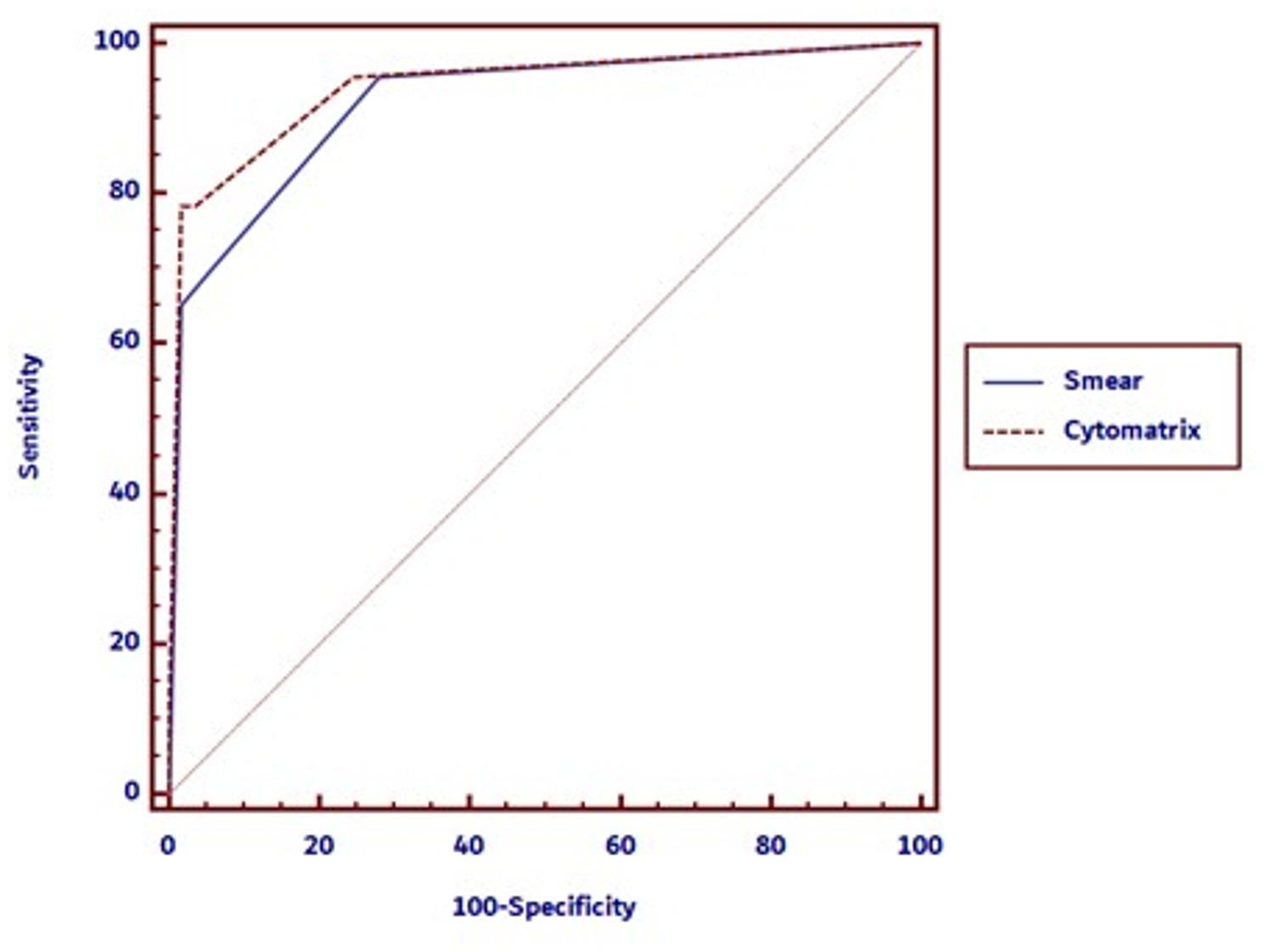
ROC curve: smear vs. cytomatrix diagnostic performance. Receiver Operating Characteristic (ROC) curve comparing Smear and Cytomatrix methods, illustrating their diagnostic performance. The dotted red line represents the Cytomatrix, while the solid blue line corresponds to the smears. The Area Under the Curve (AUC) was 0.943 for Cytomatrix and 0.921 for smears

Figure 2 displays the Receiver Operating Characteristic (ROC) curve comparing Cytomatrix and conventional smears in thyroid FNAC.

Table 5 presents the histopathological type and the corresponding number of cases for each Bethesda category in both Cytomatrix and Smears. It provides a breakdown of the Bethesda categories for each method, showing the histopathological results by type and case count.

**Table 5:** Histopathological types and case numbers for each Bethesda category in both cytomatrix and smear methods

Slide Smears	Instances		Cytomatrix	Instances
**Bethesda II**	42		**Bethesda II**	44
Nodular Hyperplasia	33		Nodular Hyperplasia	35
Basedow Disease	3		Basedow Disease	3
Chronic Thyroiditis	5		Chronic Thyroiditis	5
Papillary Thyroid Carcinoma	1		Follicular Variant of Papillary Thyroid Carcinoma	1
**Bethesda III**	19		**Bethesda III**	16
Chronic Thyroiditis	4		Chronic Thyroiditis	3
Follicular Variant of Papillary Thyroid Carcinoma	3		Follicular Variant of Papillary Thyroid Carcinoma	2
Nodular Hyperplasia	9		Nodular Hyperplasia	9
Papillary Thyroid Carcinoma	3		Papillary Thyroid Carcinoma	2
**Bethesda IV**	3		**Bethesda IV**	1
Follicular Variant of Papillary Thyroid Carcinoma	1		Nodular Hyperplasia (follicular adenoma	1
Nodular Hyperplasia (follicular adenoma)	2		**Bethesda V**	16
**Bethesda V**	16		Chronic Thyroiditis	1
Medullary Carcinoma	1		Follicular Variant of Papillary Thyroid Carcinoma	1
Nodular Hyperplasia	1		Medullary Carcinoma	1
Papillary Thyroid Carcinoma	14		Papillary Thyroid Carcinoma	13
**Total**	**80**		**Bethesda VI**	3
			Papillary Thyroid Carcinoma	3
			**Total**	**80**

**Fig. 3:**
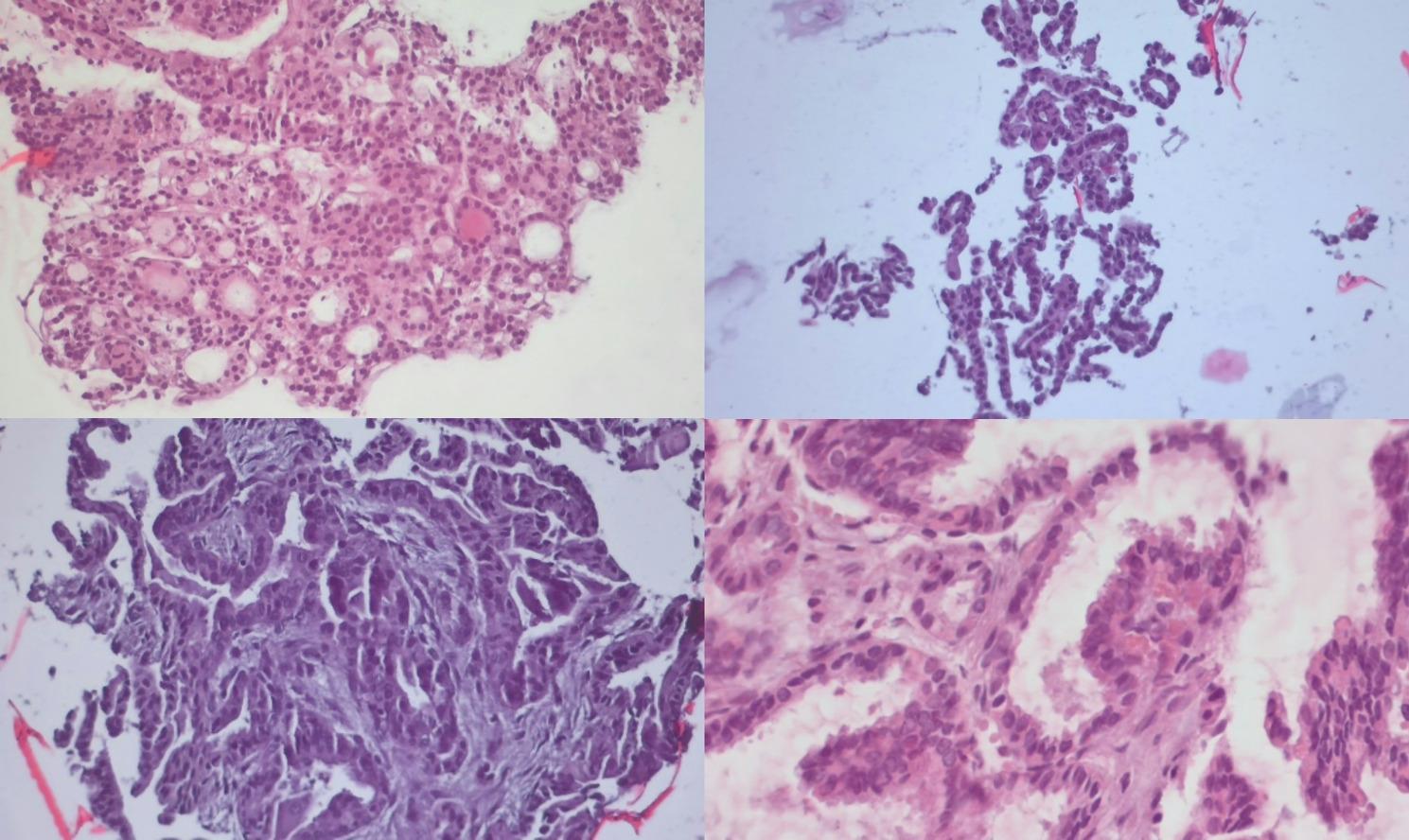
Representative image of a thyroid fine-needle aspiration sample processed with Cytomatrix, demonstrating well-preserved cellular and architectural. Thyroid lesions processed with Cytomatrix, showcasing detailed cytological characteristics at different magnifications (top left Bethesda II, top right Bethesda III, bottom left Bethesda V, bottom right Bethesda VI

Several images from different cases processed using Cytomatrix are presented in Fig. 3. These images represent a variety of thyroid lesions classified into different mirrored Bethesda risk categories, showcasing the quality and level of detail captured by Cytomatrix.

## Discussion

This study assessed the performance of Cytomatrix, an innovative cell preservation technique, against conventional smear-based cytology in thyroid fine-needle aspiration (FNAC) [12]. Cytomatrix demonstrated comparable, and in some metrics superior, diagnostic potential, offering notable advantages in sensitivity, specificity, accuracy, and sample quality.

With a concordance rate of 76.2% between Cytomatrix and smears across 80 cases, the results confirm the potential of Cytomatrix as a reliable tool in thyroid FNAC. In this cohort, Cytomatrix showed improved diagnostic parameters, particularly sensitivity (78.26% vs. 69.57%), supporting findings in previous studies that highlight its enhanced ability to detect malignancies and reduce false negatives [19, 20]. This improvement is likely attributable to superior preservation of cellular detail, enabling clearer identification of subtle malignant features often missed by smears.

Specificity was also slightly higher for Cytomatrix (96.49% vs. 94.74%), aligning with published thyroid FNAC data and suggesting a lower rate of false positives [21, 22]. The positive predictive value (PPV) for Cytomatrix (90%) exceeded that of smears (84.21%), indicating better reliability in confirming malignancy and reducing the risk of overdiagnosis and unnecessary procedures [23].

Overall diagnostic accuracy was higher for Cytomatrix (91.25% vs. 87.5%), reinforcing its value in thyroid FNAC. This improvement, along with enhanced sensitivity and PPV, likely stems from better cellular architecture preservation, which supports more precise cytological interpretation. These results are consistent with prior studies and further validate the clinical reliability of both Cytomatrix and smear-based methods in thyroid nodule assessment [24–26].

Cytomatrix’s improved sensitivity can refine thyroid FNAC workflows by reducing false negatives, enabling earlier detection of malignancies, and improving patient outcomes. Early detection is critical in preventing delayed diagnoses and disease progression [27]. Additionally, its higher specificity and PPV reduce false positives, potentially minimizing unnecessary thyroidectomies, which carry significant intraoperative risks and long-term consequences such as lifelong hormone therapy [28–30]. Overall, Cytomatrix’s enhanced diagnostic performance can optimize thyroid cytopathological evaluations, provide better treatment planning, reducing unnecessary interventions, such as unwarranted surgeries, and ensuring that patients with malignancies are identified early for timely, appropriate therapeutic actions.

In this study, Cytomatrix demonstrated the ability to both upgrade and downgrade diagnoses, improving diagnostic precision in thyroid nodule classification. It effectively upgraded cases from lower Bethesda categories, such as from Bethesda II to Bethesda V and from Bethesda III to Bethesda V, with histopathological analysis confirming malignancy in all these upgraded cases. This demonstrates superior sensitivity in detecting malignancy, particularly subtle features missed by traditional smears. Additionally, it confirmed malignancy by upgrading cases from Bethesda V to Bethesda VI, boosting diagnostic confidence. Alternatively, Cytomatrix also downgraded cases, such as from Bethesda III to Bethesda II and from Bethesda IV to Bethesda III, all of which were confirmed as benign on histopathological analysis, improving accuracy by reducing false positives and preventing overtreatment, more notably seen in the downgrade from Bethesda V to Bethesda II.

Focusing specifically on Bethesda III nodules, Cytomatrix and smears were concordant in 11 cases, with 8 proven benign and 3 malignant. Among discordant cases, Cytomatrix upgraded three Bethesda III nodules, two of which were malignant, and downgraded five to Bethesda II, with four of those confirmed benign. These findings suggest that Cytomatrix improves diagnostic accuracy by enabling both upgrades and downgrades within the Bethesda system, leading to better concordance with histopathology. By enhancing risk stratification, especially in indeterminate nodules, it supports earlier and more confident clinical decisions, potentially reducing delays in surgical management as well as unnecessary procedures such as repeat FNACs or surgeries [31].

While Cytomatrix demonstrated high sensitivity and specificity, there were a few instances where the traditional smears provided a more accurate diagnosis. For example, Cytomatrix downgraded a Bethesda III case to Bethesda II, despite the final histopathological exam revealing malignancy. In another case, a Bethesda IV diagnosis by the smears was downgraded to Bethesda III, with histopathology later classifying the case as malignant. These cases, though few in our study lot, emphasize that while Cytomatrix enhances diagnostic outcomes and can help detect malignancies that might be overlooked by conventional methods, traditional smear cytology remains a crucial reference due to its standardized and well-established guidelines [32]. This suggests that combining both methods—leveraging Cytomatrix for its improved sensitivity while relying on the smear-based method for its established accuracy, would offer the most reliable diagnostic strategy, especially in the initial stages [11, 12, 33].

Cytomatrix introduces a novel method in thyroid cytology by using a synthetic 3D structure to preserve cellular architecture, improving the use of minimal tissue samples and enhancing diagnostic efficiency. Cytomatrix reduces the need for multiple needle passes during fine needle aspiration cytology (FNAC), with minimized patient discomfort and improved diagnostic reliability [12, 34, 35]. In contrast, conventional smears, despite their safe, cost-effective, standardized nature, often compromise diagnostic accuracy due to mechanical distortion during the smearing process [11, 36]. This can lead to inconclusive or erroneous results, increasing the likelihood of false negatives. Therefore, patients may require additional FNAC procedures, prolonging diagnostic timelines, escalating healthcare costs, and adding to patient burden [30, 37]. By minimizing these challenges, Cytomatrix presents a compelling alternative for improving the efficiency and accuracy of thyroid cytology.

Two techniques were employed during the fine-needle aspiration process: the capillary method and the aspiration technique. The capillary method, which avoids aspiration, is preferred in certain cases due to its ability to minimize hemorrhagic contamination, resulting in higher-quality material, albeit with a smaller sample size. Conversely, the aspiration technique generates a larger volume of material but carries the risk of hemorrhagic contamination, which can compromise sample quality. Each method has distinct advantages, suggesting that combining both could optimize sample quality and enhance diagnostic accuracy [38–40]. Cytomatrix’s ability to preserve cellular architecture, regardless of the sampling technique, further elevates its diagnostic potential [12].

In this study, Bethesda I cases were excluded. Both Cytomatrix and the smear-based method can yield non-diagnostic results when cellular material is insufficient; however, Cytomatrix’s enhanced preservation capabilities notably reduce this risk [11, 12, 41]. The non-diagnostic rate was 9 cases for smears and 5 cases for Cytomatrix, indicating improved diagnostic adequacy when using Cytomatrix. Notably, all 5 Cytomatrix non-diagnostic cases were also non-diagnostic on smears.

The reduction in non-diagnostic cases with Cytomatrix is promising, particularly in enhancing diagnostic accuracy and reducing repeat aspirations. By preserving sample integrity with minimal cellularity, Cytomatrix improves the likelihood of obtaining a reliable diagnosis, even with limited material [12]. This is particularly beneficial in challenging cases, reducing the need for multiple needle passes and enhancing diagnostic reliability, which ultimately lessens patient burden [30, 37]. Future studies with larger cohorts are needed to confirm these findings and further assess Cytomatrix’s role in routine thyroid nodule evaluations.

A key advantage of Cytomatrix, as demonstrated in this study, is its ability to produce consistent results without the need for specialized equipment. Unlike traditional preservation methods such as cell block preparations and liquid-based cytology (LBC), which require multiple processing steps and additional resources, Cytomatrix offers a simplified and efficient alternative. Conventional cell block methods involve centrifugation, coagulation, dehydration, and embedding, increasing both time requirements and the risk of cell loss or sample distortion [42–44]. In contrast, Cytomatrix involves direct placement of the FNAC sample onto a synthetic matrix, followed by formalin fixation and standard paraffin embedding. This streamlined process eliminates the need for centrifugation or plasma-thrombin steps, reducing technical complexity while preserving cellular architecture [43]. As a result, Cytomatrix provides a cost-effective, reliable, and accessible solution, particularly advantageous in routine cytology or low-resource settings where conventional systems may be impractical [11, 12].

Another notable feature of Cytomatrix is its ability to support ancillary testing on the preserved tissue sections, ensuring their integrity for indefinite storage without compromising material quality [12]. This capability is particularly beneficial in indeterminate cases, such as the Bethesda III Category, where traditional FNAC may yield inconclusive results [45]. Cytomatrix’s ability to maintain cellular architecture, even in small or limited samples, enables the use of molecular tests. In the present study, ancillary testing was not incorporated by design, as the primary objective was to assess Cytomatrix’s diagnostic performance under standard clinical conditions, with minimal resource input and without reliance on advanced laboratory infrastructure. This approach was intended to simulate real-world settings in which ancillary tools such as molecular profiling may not be readily available. By evaluating Cytomatrix independently of supplementary techniques, we aimed to determine whether it could improve diagnostic accuracy based solely on core cytological assessment. This establishes a practical foundation for its potential utility across a wide range of clinical environments, including those with limited access to specialized diagnostics. Cytomatrix’s compatibility with molecular and immunohistochemical testing remains a key strength, with some studies already demonstrating its successful incorporation into ancillary testing workflows, further supporting its value for future clinical applications and investigations [46, 47].

When comparing Cytomatrix with other systems such as the CytoFoam Core System (CFCS), both systems rely on similar synthetic matrix technology designed to enhance cell preservation and improve diagnostic yield in thyroid FNAC. Each platform supports ancillary testing, integrates smoothly into standard histopathology workflows, and contributes to clearer classification of indeterminate cases. Both are low-cost solutions suitable for routine use, with Cytomatrix priced at approximately $13 per case and CFCS around $8 [48]. In this study, Cytomatrix was selected due to its local availability and prior evaluation in earlier research by our team. Although a direct comparison between the two systems was not conducted, their shared objectives and technical similarities suggest comparable clinical utility. Further studies may help clarify any differences in diagnostic impact, cost-efficiency, and long-term applicability in diverse clinical settings.

## Limitations and future directions

While Cytomatrix demonstrated improved diagnostic performance compared to conventional smears, certain aspects require further refinement. The low percentage of misdiagnoses is encouraging; however, it further underscores the need for more standardized interpretative criteria and a structured evaluation system, especially when applied to larger cohorts, to enhance consistency and reliability. Existing cytology guidelines may not fully capture Cytomatrix’s nuances so establishing well-defined diagnostic thresholds and guidelines could help minimize variability in interpretation, ultimately improving overall accuracy and clinical utility. A key limitation of this study is the relatively small sample size, which may limit statistical power and reduce the generalizability of the findings across broader or more diverse populations. While the 80 analyzed cases provide meaningful preliminary insights, it may not fully capture the variability seen in larger, multicenter clinical settings. Additionally, the exclusion of non-diagnostic (Bethesda I) cases, while methodologically justified for comparative accuracy analyses, further reduced the dataset size. These constraints highlight the need for larger-scale studies to confirm the observed trends and validate the technique’s reliability across a wider spectrum of clinical environments. The obtained results however lay the groundwork for future studies with larger cohorts, which will be important for further validating the technique and solidifying its role in routine cytological evaluations. Additionally, future research should standardize sample processing, evaluate observer variability, and incorporate ancillary tests to strengthen diagnostic confidence. Optimizing workflow integration and assessing cost-effectiveness will be key to maximizing Cytomatrix’s clinical impact.

## Conclusion

This study indicates that Cytomatrix is a useful tool in thyroid FNAC, with potential benefits for cytological evaluation and diagnostic performance. It showed good sensitivity, specificity, and overall accuracy, while preserving tissue architecture—an aspect that may aid in more consistent interpretation. Its role in clarifying indeterminate Bethesda III cases and highlighting subtle cytological features in higher-risk categories suggests it could assist in addressing diagnostic uncertainties. While limitations remain, Cytomatrix offers a practical approach to sample handling with minimal resource requirements and may support ancillary testing when needed. These findings support its consideration as a complementary method alongside conventional smears, particularly in diagnostically challenging cases.

## Data Availability

Data is provided within the manuscript or supplementary information files.
